# Use of Radioguided Surgery for Small and Difficult-to-Locate Relapsed MIBG (+) High-Risk Neuroblastoma Lesions

**DOI:** 10.3390/cancers16193348

**Published:** 2024-09-30

**Authors:** Lucas Krauel, Albert Pasten, Maite Gorostegui, Salvador Mañé, Marta Pilar Martin Giménez, Maria Coronas, Rosalia Carrasco Torrents, Jaume Mora

**Affiliations:** 1 Pediatric Surgical Oncology Unit, Department of Pediatric Surgery, SJD Barcelona Children’s Hospital, Universitat de Barcelona, 08950 Barcelona, Spain; apastego44@alumnes.ub.edu (A.P.); martapilar.martin@sjd.es (M.P.M.G.); maria.coronas@sjd.es (M.C.); rosalia.carrasco@sjd.es (R.C.T.); 2 Pediatric Cancer Center Barcelona (PCCB), SJD Barcelona Children’s Hospital, Universitat de Barcelona, 08950 Barcelona, Spain; maite.gorostegui@sjd.es (M.G.); jaume.mora@sjd.es (J.M.); 3 Nuclear Medicine Department, Pediatric Cancer Center Barcelona (PCCB), SJD Barcelona Children’s Hospital, Universitat de Barcelona, 08950 Barcelona, Spain

**Keywords:** high-risk neuroblastoma, MIBG, surgery, guided surgery, inmunotherapy

## Abstract

**Simple Summary:**

High-risk neuroblastoma, especially when it recurs, is challenging to treat. Since immunotherapy and target therapy have been incorporated in the treatment of these patients, tissue sample collection has a key impact on their management and prognosis. However, tissue sampling is often difficult to obtain in this population. This study investigates the use 123I-MIBG radioguided surgery, which helps surgeons access hard-to-reach areas of neuroblastoma. By using a gamma probe to detect and remove the tumor tissue, this method aims to improve tissue sampling and, ultimately, patient outcomes. The findings show that this technique was successful in all cases, allowing for complete tumor removal and enabling important molecular studies to be conducted. This approach could significantly impact how we treat and study high-risk neuroblastoma.

**Abstract:**

Introduction: High-risk neuroblastoma, particularly in the relapse/refractory (R/R) setting, poses unique challenges to obtaining the representative-quality tissue that is mostly required for molecular analysis. This study explores the use of 123I-MIBG radioguided surgery to access complex locations of MIBG-positive neuroblastoma as a tool to overcome the difficulties associated with repeated surgeries in these patients. Methods: This study is a retrospective review of all patients with R/R neuroblastoma and MIBG-uptaking lesions who underwent radioguided surgery between February 2020 and 2023 at SJD Barcelona Children’s Hospital. The Europrobe 3.2 gamma probe was used to identify neuroblastoma tissue in the operating room. Results: Ten patients were identified. Radioguided surgery was useful in all patients. One patient with previous multiple operations developed an entero-cutaneous fistula with posterior full recovery. Mean surgical time was 111.7 min. The gamma probe identified 100% of neuroblastoma lesions which were all completely removed (123I-MIBG-SPECT/CT negative post-surgery). Pathology and molecular studies could be successfully performed in all samples. Conclusions: 123I-MIBG radioguided surgery proved effective in obtaining viable tissue from difficult-to-access sites in high-risk relapsed neuroblastoma.

## 1. Introduction

Pediatric cancer has unique characteristics, which are based on a dysregulated development physiopathology. Neuroblastoma accounts for 7–10% of all solid extracranial tumors but causes an excess of 15% of all pediatric cancer deaths [1,2,3].

Neuroblastoma develops from immature nerve cells and presents a highly heterogeneous clinical spectrum. This spectrum ranges from cases that spontaneously regress without intervention to those that are aggressive and refractory to treatment, often resulting in a poor prognosis. Understanding this variability is crucial for developing effective treatment strategies.

The heterogeneity of neuroblastoma is largely influenced by the underlying biology of the tumor [4]. Key factors include genetic and molecular characteristics, such as the presence of MYCN amplification, segmental chromosomal aberrations, and mutations in genes like ALK. These biomarkers can significantly influence the tumor’s behavior and response to treatment [5].

In certain instances, neuroblastoma undergoes spontaneous regression, particularly in infants. This phenomenon, known as spontaneous differentiation or apoptosis, can occur without any therapeutic intervention and is thought to be driven by the maturation of the sympathetic nervous system and the inherent ability of the tumor cells to differentiate into benign ganglion cells.

Conversely, aggressive forms of neuroblastoma often exhibit genetic alterations that drive rapid tumor growth and resistance to conventional therapies. MYCN amplification, found in approximately 20% of cases, is one of the most potent prognostic markers for high-risk disease, correlating with rapid tumor progression, poor clinical outcomes, and relapse. Other genetic anomalies, such as 1p and 11q deletions, are also associated with unfavorable prognosis and can contribute to the complexity and aggressiveness of the disease [6].

The clinical management of neuroblastoma must therefore be tailored to the individual patient, considering the biological characteristics of the tumor. Low-risk neuroblastoma may require minimal intervention, often managed with observation or simple surgical resection [7]. In contrast, high-risk neuroblastoma entails an intensive multimodal approach, including chemotherapy, surgery, radiation therapy, immunotherapy, or targeted therapy after relapse/refractory events [8].

The addition of anti-GD2 immunotherapy to the backbone of multimodality treatment, which includes chemotherapy, surgery, and radiation, has dramatically altered the prognosis of relapse/refractory (R/R) high-risk neuroblastoma (HR-NB). The course of the disease, including the relapse pattern, has changed so much that patients may relapse multiple times, but rescue therapies allow them to survive much longer [6,9,10]. Relapses are occurring later and with lesser burden of disease [11,12]. Thus, R/R disease is becoming a complex scenario in which molecular studies can provide further understanding of the disease response, clonal evolution, and potential targets. Tissue samples in these cases are critical for diagnostic and therapeutic purposes. In this setting, surgical procedures necessary for accessing the new sites of the disease can become challenging. Patients’ previous history of repeated surgeries and radiation, as well as the small sizes and anatomical location of the new lesions, all increase the risk of complications and decrease the success rate of obtaining viable tissue [13,14].

123-iodine-metaiodobenzilguanidine (123I-MIBG) SPECT or SPECT-CT is the gold standard for imaging neuroblastoma at diagnosis and during follow-up [15,16]. MIBG is an analog of norepinephrine and enters inside the neuroendocrine cells through its specific transporter, therefore providing an excellent possibility to radio-image neuroblastoma tumors [16]. Some studies have reported the use of 123I-MIBG as a radiotracer in the surgery of neuroblastoma with an active involvement of nuclear medicine experts in the operating room [17,18,19,20]. These studies, however, were conducted at diagnosis and/or with the aim to compare performance between different radiotracers. Evidence for the use of 123I-MIBG radioguided surgery in relapse neuroblastoma is scarce. This study aimed to evaluate the efficacy of 123I-MIBG radioguided surgery in the setting of relapse neuroblastoma, particularly in complex, small, and difficult-to-locate anatomical areas.

## 2. Materials and Methods

**Figure 1:**
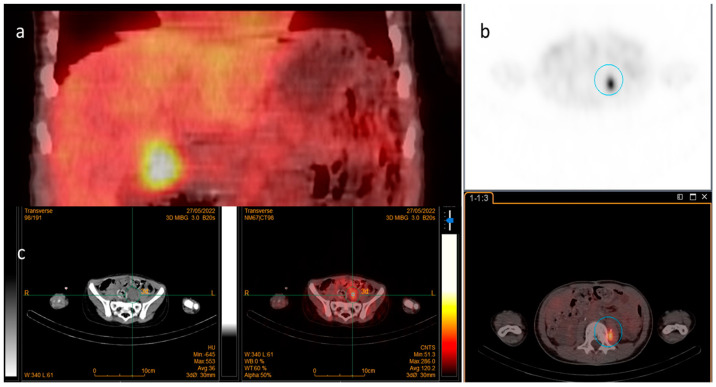
Iodine-123-MIBG SPECT CT images in three different patients with local uptake: (**a**) hepatic hilum; (**b**) left psoas muscle; (**c**) pelvic.

After IRB approval, we conducted a retrospective review of clinical records for all patients with relapsed/refractory (R/R) neuroblastoma who underwent radioguided surgery using 123I-MIBG between February 2020 and October 2023. Informed consent was obtained. This study included all patients diagnosed with high-risk neuroblastoma presenting with MIBG-avid lesions (Figure 1) who were treated with this surgical technique.

A summary of the clinical data is presented in Table 1.

In preparation for surgery, 123I-MIBG was administered via a peripheral vein 16 h prior to the procedure. We used AdreView 74 MBq/mL Injectable Solution from GE Healthcare Bio-Sciences (Chicago, IL, USA). At the calibration date and time, 1 mL of injectable solution contains 74 MBq of iobenguane (123I). The activity range per vial varies between 37 MBq and 740 MBq at the calibration date and time. The injection of AdreView should be administered intravenously and slowly over several minutes.

Iodine (123I) decays to stable tellurium (123Te) with a half-life of 13.2 h via electron capture. The most significant emitted radiation is gamma radiation of 159 KeV (with an abundance of 83.6%).

The activity to be administered to children and adolescents can be calculated according to the pediatric dose recommendations of the European Association of Nuclear Medicine (2014 EANM dosage card). Activity administered to children and adolescents can be calculated by multiplying a specific baseline activity (for calculation purposes) by the weight-determined factors indicated in the following table (Table 2).

The administration of drugs known or expected to reduce the uptake of 123I-MIBG should be interrupted before its administration (generally four biological half-lives). To minimize the radiation dose to the thyroid gland, radioactive iodine uptake should be avoided using orally administered stable iodine.

In adolescents, children, and infants, thyroid gland blocking should be performed by administering potassium iodide or potassium iodate approximately 1 h before the 123I-MIBG injection, the night of the injection day, and the following day (a total of 3 doses over 2 days). The recommended doses for thyroid gland blocking should be based on the patient’s age group (see Table 3 below).

Potassium perchlorate or sodium perchlorate can be used in patients with a history of iodine incompatibility. In children and infants, sedation may be required for SPECT imaging acquisitions. The patient should be well hydrated before the procedure begins and should be advised to urinate frequently during the first few hours after its completion to reduce radiation exposure.

The effective dose resulting from the administration of an activity (maximum recommended) of 370 MBq for a 70 kg adult is 4.8 mSv. For an administered activity of 370 MBq, the typical radiation dose to the target organ (heart) is 6.7 mGy, and the typical radiation dose to the critical organ (liver) is 24.8 mGy. Specific dosimetry can be consulted in the Supplementary Materials (Table S1).

**Figure 2:**
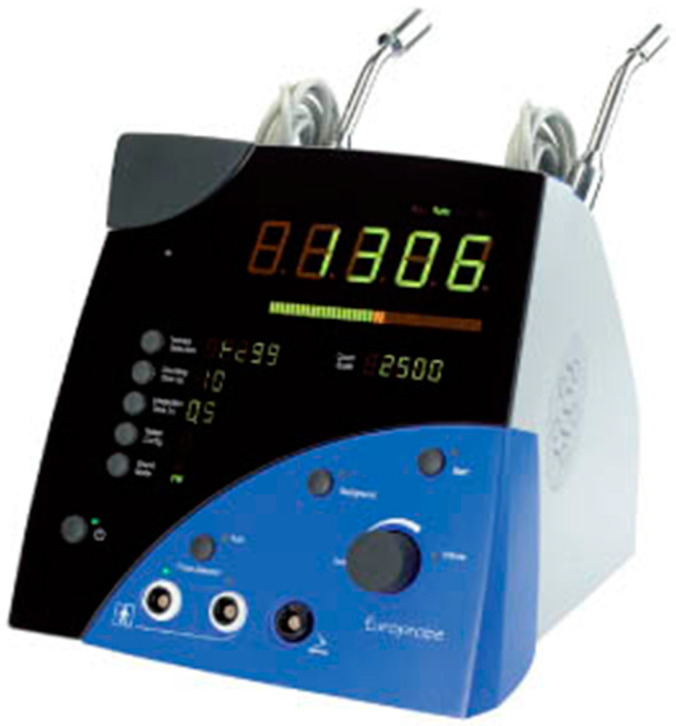
Surgical probe Eurorad^®^ Europrobe 3.2.

During surgery, a Eurorad^®^ Europrobe 3.2 radio-gamma probe (Chennevières-sur-Marne, France) (Figure 2) was utilized to detect MIBG-positive lesions. This probe is specifically designed for intraoperative use to aid in the identification of tissues emitting gamma rays. When the probe detects gamma ray emissions from the tissue, it emits a distinct pitch sound, alerting to the presence of MIBG-positive lesions. Additionally, the probe provides numerical values that indicate the intensity of the gamma radiation detected. These numerical values are always compared to those of normal tissues to accurately differentiate between healthy and MIBG-positive tissues.

**Figure 3:**
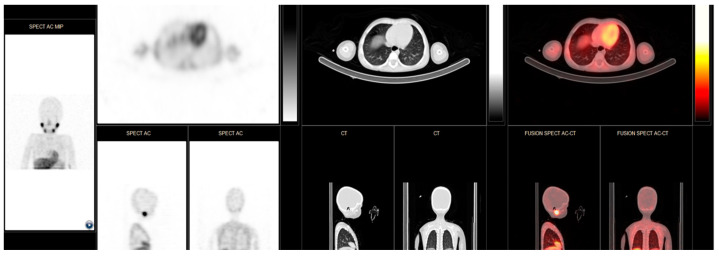
Iodine-123-MIBG SPECT CT images with normal uptake in salivary glands, heart, liver, gastrointestinal tract, and urinary system.

With the patient under general anesthesia, the probe was first tested in the suspected anatomical area before making a skin incision. The purpose of this was to establish a baseline reference count and to aid in planning the optimal surgical incision, considering previous skin incisions and the best approach based on surgical anatomy. The surgical team, including a nuclear medicine expert, then proceeded with the surgery, removing all suspected tissues under the guidance of the probe. The surgery was considered complete only when the probe indicated no gamma ray emissions from the operated area, and the numerical values returned to levels consistent with normal tissue. A key consideration during these procedures is that certain anatomical structures, including the heart, thyroid, kidneys, bladder, and ureters, naturally uptake MIBG (see Figure 3). When lesions are located near these structures, it is crucial for the nuclear medicine expert to accurately differentiate between normal physiological uptake, which is approximately 300 counts per second (cps), and neuroblastoma lesions, which typically show around 700 cps.

## 3. Results

We present a cohort of ten neuroblastoma patients (four boys and six girls), treated at a median age of 8.5 years. In all cases, the primary tumor was located in the retroperitoneum. At diagnosis, nine patients were classified as stage 4/M. Seven patients had undergone prior surgical interventions, and four had received radiotherapy. Details of MIBG-positive lesions at the time of relapse or progression are summarized in Table 4.

The majority of procedures were open surgeries (80%), with the remaining 20% performed laparoscopically. The median duration of surgery was 111 min. Lesion sizes varied, with 20% of resected lesions being less than 1 cm and 80% between 1 and 3 cm. A surgical complication was reported in one patient, who developed an entero-cutaneous fistula. This patient, a boy who had undergone four previous surgeries and received multiple rounds of radiotherapy, had the fistula managed conservatively. Closure of the fistula was achieved 4 weeks later.

Pathological examination revealed poorly differentiated neuroblastoma in 60% of cases and differentiating neuroblastoma in 40%. Molecular studies, including fluorescence in situ hybridization (FISH), next-generation sequencing (NGS), and polymerase chain reaction (PCR)-based RNA studies, were successfully conducted on all samples (Table 5). Postoperative MIBG scans were negative in all patients, indicating no residual disease.

## 4. Discussion

Since 1980, the use of radiotracers for sentinel lymph node biopsy in melanoma and other tumors has become widely spread [21,22,23], and its use is increasing with the development of new tracers [24]. Examples of new indications include parathyroid surgery and detection of lung metastasis [25,26].

Few reports have previously considered MIBG as a radiotracer for neuroblastoma surgery. Hishiki et al. described two cases in which 123I-MIBG was used for surgery [19]. Martelli and cols. conducted a comparison between two iodine radiotracers (123I versus 125I) in patients with primary tumors [20]. Works by Heij [17] and Iagaru [18] report case series of recurrent neuroblastoma surgeries using MIBG both describing positive outcomes. It is noteworthy that all these reports were published 15 or more years ago.

In the anti-GD2 immunotherapy era, patients survive longer and relapses can be successfully rescued, providing a growing complexity in the management of high-risk neuroblastoma patients. Surgeons are more frequently consulted to obtain more representative samples to help guide further treatment options. We have previously reported that anti-GD2 immunotherapy can induce differentiation in neuroblastoma cases with persistent MIBG-positive lesions [27]. Tissue sample was critical to confirm mature tissues like ganglioneuroma in these (mostly bone) anatomical locations. Moreover, loss of GD2 expression has been reported [28]; therefore, re-challenges with anti-GD2 immunotherapy benefit from continued demonstration of GD2 expression in relapsed samples [29,30]. Furthermore, up to 20% of relapsed neuroblastoma cases may acquire druggable ALK mutations [31], which is another good reason to pursue tissue procurement every time the disease progresses or recurs in order to better accomplish rescue treatment strategies.

These novel therapeutic avenues highlight the critical role of the surgical team in obtaining samples from patients who have already undergone multiple surgeries or radiation treatments. One of the primary challenges in this context is differentiating neuroblastoma tissue from necrosis, fibrosis, or normal irradiated tissues. Accurate differentiation is essential, as it significantly impacts the success of subsequent treatments.

Minimally invasive surgery introduces its own set of challenges, particularly when dealing with small lesions. Choosing the appropriate surgical approach and incision size while managing exposure is crucial. In these complex scenarios, MIBG-guided surgery proves to be an invaluable tool. It enhances the precision of locating and removing neuroblastoma tissue by providing real-time feedback on gamma ray emissions.

In previous cases, surgeons have faced significant difficulties in locating and obtaining adequate tissue samples. Differentiating neuroblastoma from other types of tissue, such as necrosis or scar tissue, is especially challenging in patients with a history of multiple prior surgeries and treatments. The presence of adhesions and extensive scar tissue further complicates the surgical field, making it harder to navigate and progress with the procedure. These factors collectively make accurate tissue sampling a complex task, crucial for effective treatment planning and subsequent therapeutic interventions. As in many neuroblastoma surgeries, the anatomical location and the presence of Image-Defined Risk Factors (IDRF) often require the specimen to be removed piecemeal [32,33]. As a result, assessing the margins becomes challenging.

Despite these challenges, we have successfully and safely obtained viable tissue samples from difficult anatomical locations, even in patients with a history of numerous prior surgeries. MIBG guidance has played a key role in facilitating the accurate acquisition of these samples, which were suitable for comprehensive molecular studies. This capability significantly increases the likelihood of identifying actionable targets and accessing potentially effective targeted therapies for these patients. The probe is user-friendly for surgeons and easy to operate in both open and minimally invasive surgeries. Regarding safety, the method poses no risk to either the surgical team or the patient, as the probe used in MIBG-guided surgery is harmless in the surgical field. The only limitation is that the lesions must be MIBG-positive for the probe to be effective.

In our series, one surgical complication occurred: an entero-cutaneous fistula in a patient with viable tumor in the hepatic hilum. The fistula resulted from an accidental section of the transverse colon during laparotomy. Although the damage was promptly repaired, the suture may have come into contact with the closed laparotomy, leading to the formation of the fistula. This complication was managed conservatively, and the fistula closed successfully after 4 weeks.

Our patient cohort exemplifies the new ‘chronic’ neuroblastoma population, which often requires recurrent biopsies and/or surgeries to identify new molecular targets. In this context, techniques such as 123I-MIBG guided surgery are highly beneficial for surgical teams, providing critical support in navigating complex cases and enhancing the precision of tissue sampling.

## 5. Conclusions

This retrospective review demonstrates that 123I-MIBG radioguided surgery is an effective technique for obtaining viable tissue from challenging locations in patients with high-risk relapsed neuroblastoma. The method proved useful in all ten patients, enabling the complete removal of neuroblastoma lesions, as confirmed by post-surgery imaging. The successful identification and removal of these lesions using the gamma probe allowed for essential pathology and molecular analyses. This technique offers a solution to the difficulties associated with repeated surgeries in these patients, potentially improving neuroblastoma outcomes and the ability to perform necessary molecular studies.

## Figures and Tables

**Table 1:** Clinical data.

Patient	Gender	Age at Diagnosis (years)	Disease Stage	Sites of Metastasis	Primary Treatment Received	Age at Relapse (years)	Sites of Relapse	Lesion Sizes
1	Boy	3.41	4/M	Lymph nodes Hepatic hilum	COJEC TVD RT SI	7.44	Hepatic hilum	1–3 cm
2	Girl	1.75	4/M	Lymph nodes Bone marrow Bone Liver	COJEC TVD PACE Irinotecan–TMZ RT SI	3.62	Inguinal	1–3 cm
3	Girl	2.41	4/M	Lymph nodes Bone marrow	SIOPEN LINES SIOP–Umbrella (referred from another center initially treated as WT) Rapid COJEC ASCT Irinotecan–TMZ RT SI	3.57	Psoas muscle	1–3 cm
4	Girl	3.66	4/M	Lymph nodes Bone marrow Bone Liver	COJEC ICE SI	9.84	Right iliac vessels	1–3 cm
5	Boy	4	4/M	Lymph nodes Bone marrow Bone	COJEC SI	4.62	Supraclavicular	<1 cm
6	Boy	5	4/M	Bone Bone marrow	CAV IFO + VP-16	5.64	Supraclavicular	1–3 cm
7	Girl	6.91	4/M	Lymph nodes Bone marrow	VCR + doxorubicin + etoposide + cisplatin RT	8.11	Supraclavicular	1–3 cm
8	Girl	4.08	4/M	bone Bone marrow	HR-NBL-1.7 SIOPEN SI	9.43	Cervical	1–3 cm
9	Girl	2.25	4/M	bone Bone marrow	Rapid COJEC Topotecan/TMZ + DB SI RT	6.72	Lung	<1 cm
10	Boy	7.41	1	-	SIOP VP-16 RT SI	14.35	Pelvic	1–3 cm

COJEC: cisplatin, vincristine, carboplatin, etoposide, and cyclophosphamide; TVD: topotecan–vincristine–doxorubicin; RT: radiotherapy; SI: surgical intervention; TMZ: temozolomide; ASCT: autologous stem cell transplant; ICE: ifosfamide, carboplatin, and etoposide; CAV: cyclophosphamide, doxorubicin, and vincristine; IFO-VP16: ifosfamide + etoposide; VCR: vincristine; DB: dinutuximab beta.

**Table 2:** A[MBq] administered = baseline activity × factor. The baseline activity is 28 MBq. The minimum recommended activity is 37 MBq.

Weight (kg)	Factor	Weight (kg)	Factor	Weight (kg)	Factor
3	1	22	5.29	42	9.14
4	1.14	24	5.71	44	9.57
6	1.71	26	6.14	46	10.00
8	2.14	28	6.43	48	10.29
10	2.71	30	6.86	50	10.71
12	3.14	32	7.29	52–54	11.29
14	3.57	34	7.72	56–58	12.00
16	4.00	36	8	60–62	12.71
18	4.43	38	8.43	64–66	13.43
20	4.86	40	8.86	68	14.00

**Table 3:** Recommended doses for thyroid blocking in infants, children, adolescents, and adults.

Patient Age Group	Potassium Iodide (mg)	Potassium Iodate (mg)
Infants (1 month–3 years) *	32	42
Children (3–12 years) *	65	85
Adolescents (>12 years) *	130	170
Adults **	130	170

* Three administrations are needed over 2 days. ** A single administration is necessary.

**Table 4:** Locations, number, and surgical approach of resected lesions with aid of MIBG.

Location of MIBG (+) Lesions	N/%	Surgical Approach
Right iliac vessels	1/10% each	Minimally invasive
Hepatic hilum		
Cervical		Open
Inguinal		
Pelvic		
Lung		
Psoas muscle		
Supraclavicular	3/30%	

**Table 5:** Molecular and pathological findings at diagnosis and relapse.

Patient	Molecular Findings at Diagnosis	Molecular Findings at Relapse	Pathological Result
1	MYCN-NA DNA diploid	GD2 + ALK - P53 -	PDN
2	MYCN-NA Heterozygous deletion in 3p26.3 ALK -	PHOX2B + ALK - GD2 +	DN
3	MYCN-A GD2+	PHOX2B + ALK - GD2 + p53 + PDL1 - PARP1 + Bcl2 + SPARC +	PDN
4	N/A	PHOX2B + ALK - p53 -	DN
5	MYCN-NA PHOX2B + CD56+ Ki 67 +	MYCN-NA CHD5 + GD2 +	DN
6	MYCN-NA 11q deletion Synaptophysin + PHOX2B + S100 + ALK - p53 - Ki67 70%	Synaptophysin + PHOX2B + ALK - Ki67 <5% GD2 +	DN
7	MYCN-NA	Synaptophysin + PHOX2B - ALK - P53 - Ki67 2%	PDN
8	MYCN-NA 11q deletion Chromogranin + Synaptophysin + Ki67 79%	PHOX2B +	PDN
9	N/A	PHOX2B + Synaptophysin + CHD5 + ATRX + GD2 + ALK - pc53 -	PDN
10	NMYC-NA 11q deletion	PHOX2B + GD2 + ALK - PARP1 + SPARC +	PDN

PDN: poorly differentiated neuroblastoma; DN: differentiating neuroblastoma; MYCN-A: MYCN amplified; MYCN-NA: MYCN non-amplified.

## Data Availability

Data sharing is not applicable to this article as no new data were created or analyzed in this study.

## Supplementary Materials

The following supporting information can be downloaded at: https://www.mdpi.com/article/10.3390/cancers16193348/s1, Table S1. The table displays the internal radiation dosimetry calculated according to publication No. 80 of the International Commission on Radiological Protection (ICRP).
