# Supplementary material for: Use of Radioguided Surgery for Small and Difficult-to-Locate Relapsed MIBG (+) High-Risk Neuroblastoma Lesions
Source: Cancers (Basel). 2024 Sep 30;16(19):3348. doi: 10.3390/cancers16193348 (PMC11475745; doi:10.3390/cancers16193348)
Supplement: Supplementary file 1 [file cancers-16-03348-s001.zip › cancers-3137890-supplementary.pdf]

Supplementary Materials
DOSIMETRY

Table S1. The following table displays the internal radiation dosimetry calculated according to publication No. 80 of the International Commission on Radiological Protection (ICRP).

Organ	Absorbed Dose per Unit of Administered Activity (mGy/MBq				
	Adult	15y	10y	5y	1y
Adrenal Glands	0.017	0.022	0.032	0.045	0.071
Bladder	0.048	0.061	0.078	0.084	0.15
Bone Surfaces	0.011	0.014	0.022	0.034	0.068
Brain	0.0047	0.006	0.0099	0.016	0.029
Breast	0.0053	0.0068	0.011	0.017	0.032
Gallbladder	0.021	0.025	0.036	0.054	0.01
Stomach	0.0084	0.011	0.019	0.03	0.056
Small Intestine	0.0084	0.011	0.018	0.028	0.051
Ascending Colon	0.0091	0.012	0.02	0.033	0.058
Descending Colon	0.0079	0.01	0.016	0.023	0.043
Heart	0.018	0.024	0.036	0.055	0.097
Kidneys	0.14	0.017	0.025	0.036	0.061
Liver	0.067	0.087	0.13	0.18	0.33
Lungs	0.016	0.023	0.033	0.049	0.092
Muscle	0.0066	0.0088	0.013	0.021	0.037
Esophagus	0.0068	0.0088	0.013	0.021	0.037
Ovaries	0.0082	0.011	0.016	0.025	0.046
Pancreas	0.013	0.017	0.026	0.042	0.074
Bone Marrow	0.0064	0.0079	0.012	0.018	0.032
Skin	0.0042	0.0051	0.0082	0.013	0.025
Spleen	0.02	0.028	0.043	0.066	0.12
Testicles	0.0057	0.0088	0.013	0.021	0.037
Thymus	0.0068	0.0088	0.013	0.021	0.037

Thyroid	0.0056	0.0073	0.012	0.019	0.036
Uterus	0.01	0.013	0.02	0.029	0.053
Remaining Body	0.0067	0.0085	0.013	0.02	0.037
**Effective dose (mSv/MBq)**	**0.013**	**0.017**	**0.026**	**0.037**	**0.068**

The effective dose resulting from the administration of a maximum recommended activity of 370 MBq for a 70 kg adult is 4.8 mSv. For an administered activity of 370 MBq, the typical radiation dose to the target organ (heart) is 6.7 mGy, and the typical radiation dose to the critical organ (liver) is 24.8 mGy.
